# Monocyte-to-lymphocyte ratio affects prognosis in LAA-type stroke patients

**DOI:** 10.1016/j.heliyon.2022.e10948

**Published:** 2022-10-04

**Authors:** Cheng-ju Wang, Chun-yang Pang, Huan-Yu, Yi-fan Cheng, Hong Wang, Bin-bin Deng, Huan-jie Huang

**Affiliations:** The First Affiliated Hospital of Wenzhou Medical University, Wenzhou, China

**Keywords:** Acute ischemic stroke (AIS), Large artery atherosclerotic stroke (LAA), Monocytes to lymphocytes ratio (MLR), Inflammation

## Abstract

Nowadays, the prognostic prediction of acute ischemic stroke (AIS) patients is still challenging because of the limited predictive properties of existing models. Blood-based biomarkers may provide additional information to the established prognostic factors. Markers of atherosclerosis have been identified as one of the most promising biomarkers for predicting prognosis, and inflammation, in turn, affects atherosclerosis. According to previous studies, the ratio of monocytes to lymphocytes (MLR) has been reported as a novel indicator of inflammation. Thus, our study was the first to conduct more in-depth research on the relationship between MLR and the prognosis of large artery atherosclerosis (LAA)-type AIS patients.

A total of 296 patients with LAA-type stroke were recruited. Of these, 202 patients were assigned to the development cohort, and 94 patients were assigned to the validation cohort. In the development cohort, 202 patients were divided into groups A, B, C, and D according to the quartile method of MLR levels. The one-year prognosis of patients was tracked, and the modified Rankin scale (MRS, with a score ranging from 0 to 6) was mainly selected as the measurement result of the function. The relationship between MLR and prognosis was analyzed by building logistics regression models. The models showed that MLR made significant predictions in poor outcomes of LAA-type stroke patients (odds ratio: 4.037; p = 0.048). At the same time, receiver operating characteristics (ROC) curves were used to compare the predictive values between MLR and clinical prediction score (Barthel Index).

This study demonstrated that patients with LAA-type stroke and high MLR had a poor prognosis. MLR might be a reliable, inexpensive, and novel predictor of LAA-type stroke prognosis.

## 1 Introduction

Acute ischemic stroke (AIS) is a syndrome of cerebral ischemia caused by a local blood supply disorder and necrosis due to hypoxia, resulting in corresponding symptoms of a functional brain defect [1]. Among the various AIS subtypes in the classification system of stroke treatment (TOAST) [2], atherosclerosis of large arteries (LAA) is a key subtype. It is very harmful to human health [3] and increases the economic and social burdens. Therefore, it is of great significance to clarify the pathogenesis and pathological manifestations of LAA-type stroke, which can greatly help in its prognosis and treatment.

At present, many studies have shown that atherosclerosis is considered one of the main mechanisms of LAA stroke. When the arterial plaque is grown to a certain extent, it will rupture and bleed, and the fragments inside the plaque will enter the cerebral vessels with the blood flow, which will lead to hypoxia in brain tissue and cause LAA cerebral infarction. In the pathogenesis of atherosclerosis in acute myocardial infarction (AMI), inflammation and immune dysfunction play a major role [4]. Inflammation regulated by immune cells can accelerate the progression of atherosclerosis and lead to plaque rupture. Meanwhile, the secondary progression of neuronal injury occurs by the infiltration of immune cells and the release of pro-inflammatory cytokines. It is also will increase the destruction of the blood-brain barrier and infarct volume and trigger a series of AIS-related serious adverse events [5].

Increased monocytes play a significant role in AMI and participate in all stages of atherosclerosis development [6, 7, 8]. Lymphocytes characterize the immune response in the body, and a decrease in their number often indicates an inflammatory response in the body. Studies have shown that the decrease of lymphocytes is inversely proportional to the occurrence of atherosclerosis, and the lower lymphocyte count is considered to be independently related to the increase in the probability of dysfunctional outcomes [9]. It has been reported that monocyte/lymphocyte ratio (MLR) is a new inflammatory marker and a prognostic indicator [10, 11, 12]. MLR is more stable and has more predictive value than a single index.

Atherosclerotic vascular conditions are associated with increased inflammation. On the other hand, MLR has been introduced as a marker of inflammation in various conditions, such as acute coronary syndrome [13], cancer [14], COVID-19 [15], and tuberculous pleuritis [16]. Similar to ischemic stroke, all these conditions are associated with inflammation. Therefore, serum MLR seems to be related to the neurological prognosis of LAA-type stroke. At present, the modified Rankin scale (MRS) and Barthel index (BI) are commonly used in clinical practice to predict the prognosis of cerebral infarction. Previous studies have found that MRS is sensitive and responsive in measuring stroke disability compared with BI [17]. Therefore, in our study, MRS was considered the main prognostic indicator, while BI had strong subjectivity in predicting prognosis, suggesting that we might need a more objective and simpler indicator. MLR, an inflammatory index characterizing atherosclerosis, seems to be a very good choice. At present, as no one has studied the predictive relationship between MLR and LAA-type cerebral infarction, we performed such a study.

## 2 Materials and methods

### 2.1 Patient selection

From April 2018 to July 2021 at the First Affiliated Hospital of Wenzhou Medical University, 390 AIS patients were screened and participated in this retrospective research. Ischemic stroke was defined by the definition of the World Health Organization (WHO) [18], and magnetic resonance imaging (MRI) scans and brain computed tomography (CT) were used to diagnose all hospitalized patients. The National Institutes of Health Stroke Scale (NIHSS) and Alberta Stroke Program Early CT Score (ASPECTS) [19] were used to determine the severity of the nervous system damage at admission. According to the TOAST classification, stroke subtypes included LAA, small-vessel occlusion, cardiogenic embolism, and others. LAA-type patients who met TOAST criteria were included in this study.

**Figure 1:**
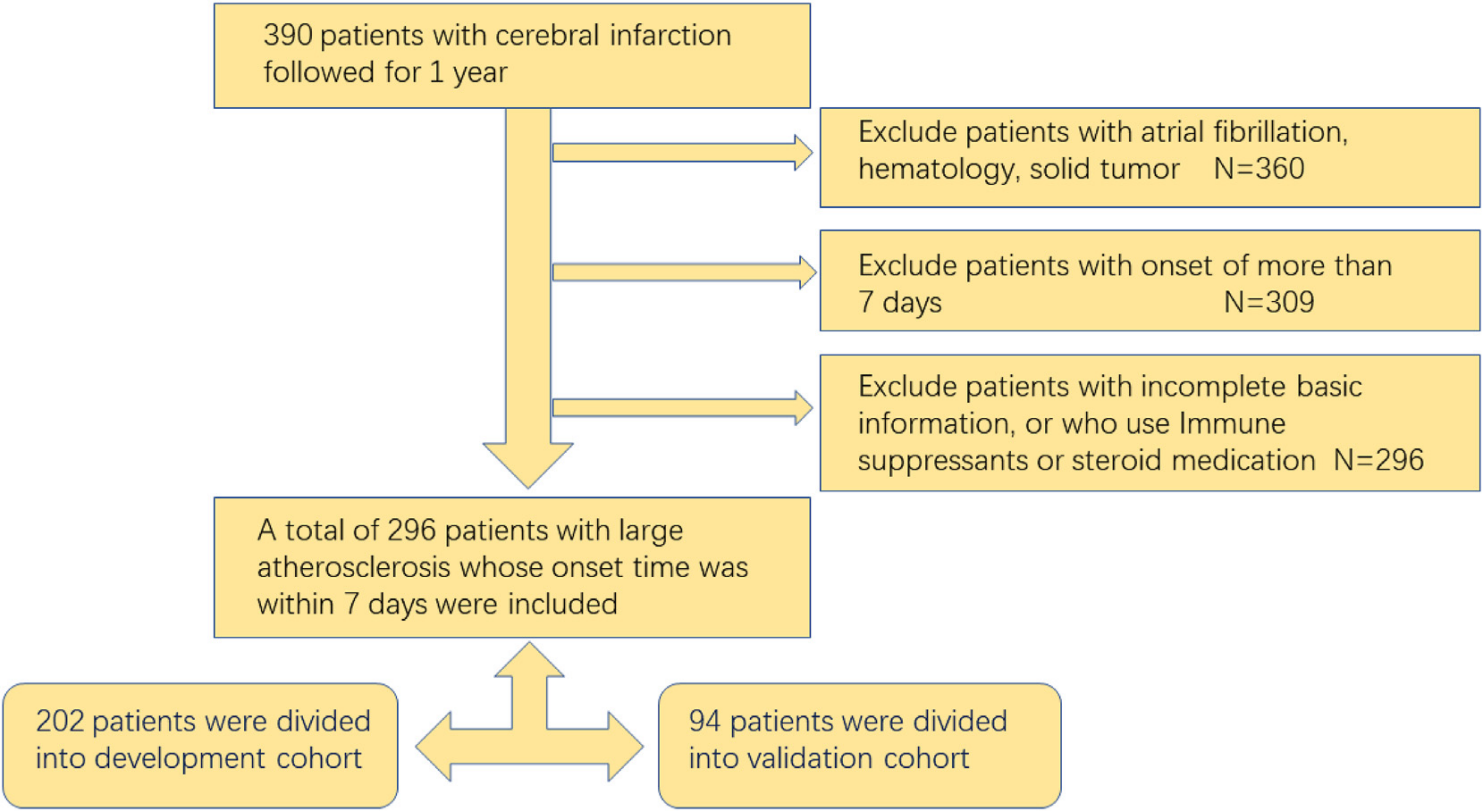
Study flow chart.

Exclusion criteria comprised (1) the time of onset exceeding seven days; (2) other existing cerebral infarction; (3) presence of other unrelated brain diseases, severe lung diseases, or heart diseases; (4) presence of autoimmune diseases, cancer, and hematologic disorders; (5) patients taking immune suppressants or steroid medication; (6) patients with unsatisfactory follow-up (refusing to follow-up or lost to follow-up); (7) patients without complete clinical data. The Medical Ethics Committee of the First Affiliated Hospital of Wenzhou Medical University approved this research (Figure 1).

### 2.2 Data collection

The recorded basic information and clinical features for the patients included age, gender, time from onset to admission, and a history of hypertension, coronary heart disease (CHD), diabetes, atrial fibrillation, diabetes, stroke, hyperlipidemia, smoking, and alcohol consumption. On the morning of the second day after admission, patients were assessed with blood indexes in a fasting state. The obtained parameters included counts of white blood cells (WBCs), red blood cells, platelets, lymphocytes, and monocytes and levels of hemoglobin, fasting blood glucose (FBG), albumin, aspartate aminotransferase, alanine aminotransferase, serum creatinine, thyroxine, triglycerides (TG), total cholesterol (TC), low-density lipoprotein cholesterol, and high-density lipoprotein cholesterol. The MLR was defined by monocyte and lymphocyte counts. A CT was completed within 24 h of admission, and an MRI was performed during hospitalization. Given that previous research contained about 2/3–4/5 people as the development group, which looks more suitable and common [20, 21], in our study, we applied SPSS to stochastically assign about 2/3 of the people to the development cohort, while the rest was assigned to the validation cohort. We divided the patients in the development cohort into four groups by ordering from high to low MLR, thereby obtaining 51, 49, 52, and 50 patients in these groups. The groups were named group A, group B, group C, and group D.

### 2.3 Evaluation of prognosis

The severity of stroke at admission was evaluated according to the NIHSS. In previous studies, an NIHSS score of ≥6 defined severe stroke, while a score of ≤6 indicated mild stroke [22]. The functional results were measured with the modified Rankin scale (MRS, with a score ranging from 0 to 6) and BI (with a score ranging from 0 to 100) one year after stroke [23, 24] since these are the most common scales for assessing stroke outcomes in clinical practice and trials. In our study, MRS was used as the primary prognostic rating scale, and BI was the secondary prognostic rating scale. Based on previous studies, good function result was defined as 0–2 points, and poor function result was defined as 3–6 points [25]. Additionally, the study participants were classified as extremely dependent (0–20), severe dependent (25–40), moderate dependent (45–60), mild dependent (65–80), and not dependent (85–100) using the BI [26].

### 2.4 Statistical analysis

We applied the SPSS 26.0 to analyze the data. All variables were tested for normality using the Kolmogorov-Smirnov test, and homogeneity of variance was also analyzed. LAA-type patients were divided into four groups based on MLR values. For the comparison of variables between the two groups, the student test and Mann-Whitney U test were applied for continuous variables, and the chi-square test was employed for classified variables. In multiple group comparisons, mean and standard deviation were summarized in the continuous variables, and frequency (%) was expressed in categorical variables. Group comparisons were performed using variance analysis of Wilcoxon rank-sum test results for continuous variables, and categorical variables adopted the chi-square test. The relationship between MLR and neurological dysfunction and relationships between other factors and MLR were analyzed by Pearson correlation analysis. The area under the receiver operating characteristic curve (ROC) was applied to evaluate the ability of MLR predictions and calculate cutoff points. Binary logistic regression analysis was used to analyze the possible risk factors for neurological impairment and clinical outcomes, followed by the estimation of multivariate-adjusted odds ratios (ORs) and 95% confidence intervals (CIs). Furthermore, age, dyslipidemia, and albumin levels were included in the multivariate models. ROC curve was used for prediction analysis of the comparison between models and BI. A P of <0.05 was considered statistically significant.

## 3 Results

### 3.1 Baseline characteristics and laboratory parameters of research subjects

The study cohort included 296 patients with LAA according to inclusion criteria. Thirty patients were excluded because they did not have LAA-type stroke, 51 patients were excluded because they were hospitalized for more than one week, and 13 patients were excluded due to loss of follow-up or lack of complete clinical data. Finally, we enrolled 296 LAA cerebral infarction patients in this research and put them into two groups. A total of 202 patients were assigned to the development cohort, and 94 patients were assigned to the validation cohort. We divided the 1-year prognosis into good outcomes and bad outcomes according to MRS level and compared the basic information between the total and development cohorts. We found that NIHSS, ASPECTS, history of hyperlipidemia, history of hypertension, neutrophil count, monocyte count, lymphocyte count, albumin level, and MLR were different between the two groups (Table 1). In the development cohort, LAA was divided into four groups based on MLR values: 51 cases (25.2%) in group A (MLR <0.217), 49 cases (24.3%) in group B (MLR 0.217–0.297), 52 cases (25.7%) in group C (MLR 0.298–0.420), and 50 patients (24.7%) in group D (MLR >0.420). Patients in group 4 had significantly increased WBC and monocyte counts and MLR. Additionally, in other groups, the number of lymphocytes was higher than in group 4 (Table 2).

**Table 1:** The general characteristics of the total and development cohorts.

	Total			Development cohort		
	Good	Poor	P	Good	Poor	P
N of patients	229	67		153	49	
NIHSS	2.82 ± 2.10	5.63 ± 2.91	<0.001∗	2.71 ± 1.69	6.10 ± 2.81	<0.001∗
ASPECTS	7.72 ± 1.16	6.75 ± 1.69	<0.001∗	7.67 ± 1.15	6.31 ± 1.65	<0.001∗
BI of 1 year	99.78 ± 1.22	67.09 ± 21.66	<0.001∗	99.87 ± 0.80	67.65 ± 22.06	<0.001∗
HL n (%)	131 (57.2%)	20 (29.9%)	<0.001∗	78 (51.0%)	15 (30.6%)	0.013∗
Smoke n (%)	99 (43.2%)	31 (46.3%)	0.659	61 (39.9%)	22 (44.9%)	0.533
Drink, n (%)	84 (36.7%)	23 (34.3%)	0.724	51 (33.3%)	14 (28.6%)	0.535
HP, n (%)	189 (82.5%)	62 (92.5%)	0.045∗	125 (81.7%)	46 (93.9%)	0.040∗
DM, n (%)	89 (39.0%)	28 (41.8%)	0.685	57 (37.3%)	22 (44.9%)	0.340
SBP	155.10 ± 22.09	157.18 ± 17.72	0.426	155.31 ± 21.77	157.24 ± 19.00	0.577
DBP	82.78 ± 13.36	80.39 ± 11.70	0.186	82.54c13.17	81.47 ± 12.06	0.613
RBC	4.74 ± 2.70	4.47 ± 0.41	0.417	4.79 ± 3.28	4.47 ± 0.44	0.507
HB	139.32 ± 18.30	133.85 ± 14.31	0.025∗	137.84 ± 19.09	133.41 ± 15.14	0.140
Platelet	222.91 ± 61.61	213.30 ± 62.42	0.264	226.07 ± 57.04	221.96 ± 63.70	0.670
WBC	6.55 ± 1.79	7.00 ± 2.23	0.093	6.44 ± 1.73	7.18 ± 2.42	0.020∗
Neutrophil	3.99 ± 1.66	4.70 ± 2.09	0.004∗	3.91 ± 1.62	4.94 ± 2.31	0.001∗
Monocyte	0.51 ± 0.17	0.61 ± 0.25	<0.001∗	0.51 ± 0.17	0.62 ± 0.27	0.001∗
Lymphocyte	1.86 ± 0.66	1.56 ± 0.51	0.001∗	1.83 ± 0.70	1.50 ± 0.50	<0.001∗
Albumin	38.74 ± 3.50	37.09 ± 3.64	0.001∗	38.38 ± 3.17	36.99 ± 3.57	0.010∗
ALT	22.56 ± 14.42	20.70 ± 15.23	0.359	21.77 ± 14.28	20.27 ± 15.93	0.533
AST	23.54 ± 9.34	24.16 ± 10.84	0.644	23.39 ± 9.17	23.76 ± 11.38	0.821
Creatinine	73.47 ± 38.27	73.34 ± 24.32	0.980	76.01 ± 45.57	75.10 ± 26.86	0.895
TC	4.83 ± 1.26	4.48 ± 0.83	0.009∗	4.88 ± 1.25	4.53 ± 0.78	0.024∗
TG	1.90 ± 1.04	3.41 ± 16.45	0.455	1.96 ± 1.12	4.14 ± 19.23	0.432
LDL	2.75 ± 0.88	2.62 ± 0.67	0.221	2.72 ± 0.82	2.67 ± 0.65	0.663
HDL	1.12 ± 0.33	1.12 ± 0.25	0.977	1.14 ± 0.37	1.11 ± 0.25	0.697
FBG	6.02 ± 2.35	5.61 ± 1.57	0.096	6.15 ± 2.47	5.77 ± 1.73	0.308
Thyroid	103.40 ± 19.14	106.53 ± 19.27	0.244	103.04 ± 18.97	107.78 ± 18.95	0.131
MLR	0.31 ± 0.24	0.42 ± 0.20	0.001∗	0.33 ± 0.28	0.45 ± 0.21	0.006∗
NLR	2.62 ± 2.95	3.39 ± 2.35	0.050	2.76 ± 3.49	3.74 ± 2.63	0.073

Abbreviation: BI: Barthel Index; HL: Hyperlipidemia; HP: hypertension; DM: diabetes mellitus; NIHSS: the National Institutes of Health Stroke Scale; SBP: systolic pressure; DBP: diastolic pressure; RBC: red blood cell; HB: hemoglobin; WBC: white blood cell; ALT: alanine aminotransferase; AST: aspartate aminotransferase; TC: total cholesterol; TG: triglyceride; LDL: low density lipoprotein; HDL: high-density lipoprotein; FBG: fasting blood glucose; MLR: monocyte-to-lymphocytes ratio. NLR: neutrophil -to-lymphocytes ratio (∗p < 0.05).

**Table 2:** Characteristics of the development cohort by Monocyte-to-Lymphocytes levels.

Characteristics	Monocyte-to-Lymphocyte ratio (MLR)				P
	Group A ＜0.217	Group B 0.217–0.297	Group C 0.298–0.420	Group D >0.420	
N of patients	51	49	52	50	
NIHSS	2.69 ± 0.26	3.14 ± 0.33	3.54 ± 0.30	4.76 ± 0.43	<0.001∗
MRS of 1 year	1.22 ± 0.12	1.50 ± 0.17	1.78 ± 0.16	2.32 ± 0.22	0.002∗
BI of 1 year	98.53 ± 4.51	93.78 ± 18.70	92.88 ± 13.44	82.90 ± 24.10	<0.001∗
ASPECTS	7.82 ± 0.87	7.45 ± 1.43	7.44 ± 1.16	6.64 ± 1.79	<0.001∗
Age, y	60.53 ± 1.7	64.54 ± 1.5	65.29 ± 1.7	68.85 ± 1.6	0.003∗
Gender, n (%)	22 (43.1%)	25 (51.0%)	41 (78.8%)	40 (80%)	<0.001∗
DM, n (%)	20 (39.2%)	18 (36.7%)	20 (38.5%)	21 (42%)	0.960
HL, n (%)	29 (56.9%)	24 (49.0%)	20 (38.5%)	20 (40%)	0.211
Smoking, n (%)	13 (25.5%)	20 (40.8%)	28 (53.8%)	22 (44%)	0.032∗
Drinking, n (%)	10 (19.6%)	17 (34.7%)	21 (40.4%)	17 (34%)	<0.001∗
SBP	154.08 ± 2.9	150.67 ± 2.9	158.51 ± 2.6	158.96 ± 3.3	0.180
DBP	83.24 ± 1.9	81.83 ± 1.8	83.9 ± 1.7	81.17 ± 1.8	0.803
RBC	4.52 ± 0.09	5.30 ± 0.82	4.53 ± 0.08	4.49 ± 0.09	0.906
HB	136.2 ± 2.48	137.73 ± 2.61	136.98 ± 2.37	135.26 ± 2.82	0.958
Palate	231.86 ± 8.75	230.31 ± 7.52	229.12 ± 8.51	210.56 ± 7.96	0.239
WBC	6.36 ± 0.19	6.13 ± 0.19	6.45 ± 0.24	7.58 ± 0.40	0.010∗
Monocyte	0.40 ± 0.019	0.49 ± 0.018	0.55 ± 0.020	0.70 ± 0.038	<0.001∗
Lymphocyte	2.36 ± 0.09	1.89 ± 0.08	1.57 ± 0.06	1.18 ± 0.06	<0.001∗
Albumin	38.88 ± 0.48	38.03 ± 0.41	37.90 ± 0.49	37.36 ± 0.50	0.146
ALT	23.51 ± 2.56	23.00 ± 2.36	19.39 ± 1.39	19.40 ± 1.737	0.440
AST	22.86 ± 1.600	23.10 ± 1.143	23.78 ± 1.39	23.84 ± 1.30	0.602
creatinine	69.22 ± 2.34	71.14 ± 2.94	73.86 ± 2.74	88.78 ± 10.78	0.155
TC	5.14 ± 0.16	4.75 ± 0.13	4.74 ± 0.19	4.54 ± 0.15	0.066
TG	2.26 ± 0.19	1.96 ± 0.16	4.31 ± 2.74	1.52 ± 0.10	0.003∗
LDL	2.91 ± 0.12	2.61 ± 0.10	2.70 ± 0.12	2.60 ± 0.95	0.140
HDL	1.15 ± 0.06	1.17 ± 0.47	1.07 ± 0.03	1.13 ± 0.06	0.630
FBG	6.13 ± 0.37	6.05 ± 0.26	5.69 ± 0.28	6.38 ± 0.38	0.163
Thyroid	101.1 ± 2.39	103.7 ± 2.43	106.1 ± 2.38	106.3 ± 3.60	0.452
MLR	0.173 ± 0.004	0.258 ± 0.003	0.355 ± 0.005	0.654 ± 0.06	<0.001∗
NLR	1.526 ± 0.458	1.96 ± 0.998	2.82 ± 0.934	5.680 ± 0.813	<0.001∗

Abbreviation: NIHSS: the National Institutes of Health Stroke Scale; MRS: modified Rankin scale; BI: Barthel Index; HL: Hyperlipidemia; DM: diabetes mellitus; SBP: systolic pressure; DBP: diastolic pressure; RBC: red blood cell; HB: hemoglobin; WBC: white blood cell; ALT: alanine aminotransferase; AST: aspartate aminotransferase; TC: total cholesterol; TG: triglyceride; LDL: low density lipoprotein; HDL: high-density lipoprotein; FBG: fasting blood glucose; MLR: monocyte-to-lymphocytes ratio; NLR: neutrophil -to-lymphocytes ratio. (∗p < 0.05).

### 3.2 The relationship between MLR and stroke severity and prognosis

**Figure 2:**
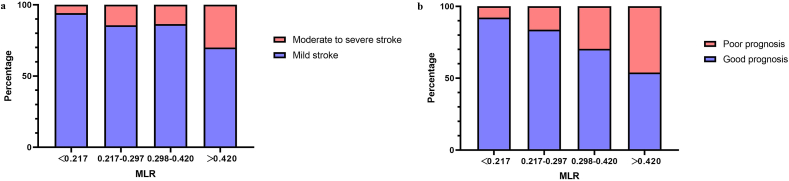
a. The severity of LAA patients based on MLR b. The prognosis of LAA patients based on MLR.

We divided the patients in the development cohort into four groups by using the MLR quartering method to further explore the relationship between MLR and disease severity and disease prognosis in patients with LAA. More than 30% of patients who had high MLR (MLR >0.420) were experiencing moderate to severe neurological deficits (Table 3). However, with the decrease in MLR, this proportion decreased (13.5%, 14.3%, and 5.88%, respectively) (Figure 2a). Undeniably, as MLR increased, the number of poor prognoses increased (P < 0.001). Additionally, 46.0% of patients in group D with high MLR (MLR >0.420) continued to have adverse outcomes, compared to 26.0% and 16.3% in groups B and C with medium MLR (0.217 ≤ MLR ≤0.297 and 0.298 ≤ MLR ≤0.420). In contrast, group A with low MLR (MLR <0.217) had a lower incidence of adverse outcomes of 7.84% (Figure 2b). It had a directed relationship between MLR and prognosis by calculating the distribution of MRS scores in the four groups. As shown, patients with higher MLR in group 4 had higher MRS scores and more moderate to severe strokes compared to the other three groups (Table 3).

**Table 3:** Comparison of outcomes among subgroups based on MLR in development cohort.

Outcomes	Total	MLR on admission			
		A < 0.217	B:0.217–0.297	C:0.298–0.420	D > 0.420
		51 (25.2%)	49 (24.3%)	52 (25.7%)	50 (24.7%)
Moderate to severe stroke (NIHSS score >5) (%)	32 (15.8)	3 (5.88)	7 (14.3)	7 (13.5)	15 (30.0)
Poor outcome (MRS score ≥3) (%)	49 (24.2)	4 (7.84)	8 (16.3)	14 (26.9)	23 (46.0)
Moderate to severe stroke with poor outcome (%)	27 (13.3)	2 (3.92)	5 (10.2)	7 (13.5)	13 (26.0)
Mild stroke with poor outcome (%)	22 (10.9)	2 (3.92)	3 (6.12)	7 (13.5)	10 (20.0)

Abbreviation: NIHSS: the National Institutes of Health Stroke Scale; MRS: modified Rankin scale.

### 3.3 Independent risk predictors and model analysis in LAA

**Figure 3:**
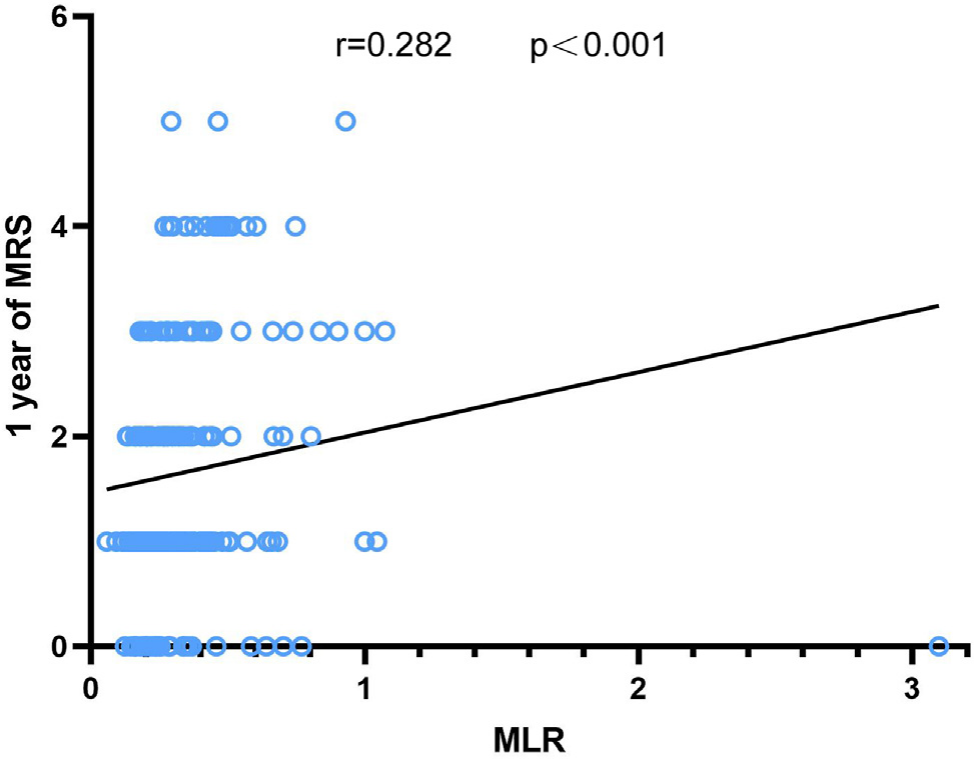
The correlation between monocyte-to-lymphocyte ratio (MLR) and 1-year MRS MLR, monocyte-to-lymphocyte ratio; MRS, modified Rankin scale.

**Figure 4:**
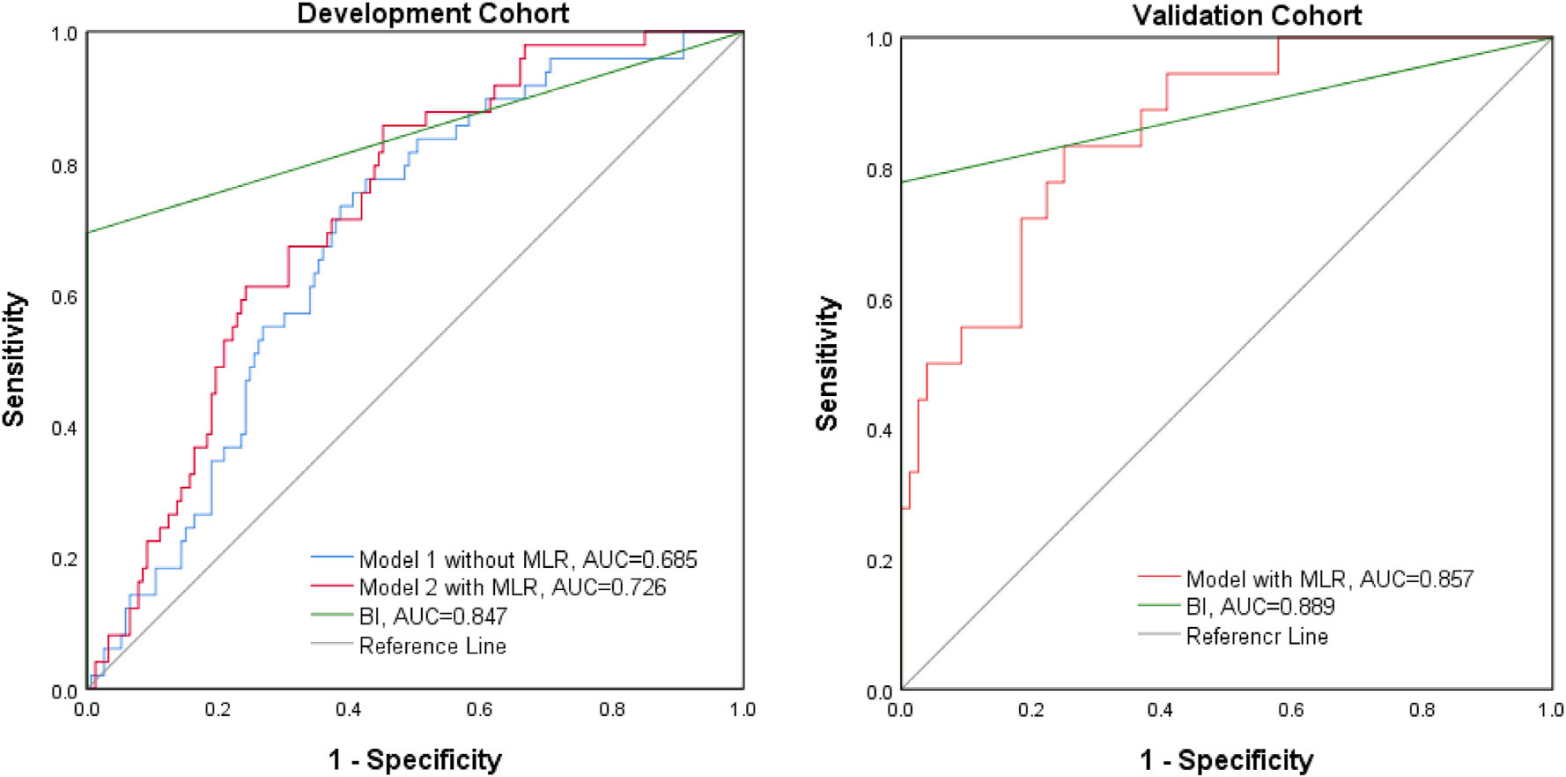
The area under the ROC curve of the prediction model of the development and validation cohorts as compared to the clinical prediction score BI, Barthel Index.

Figure 3 clearly shows a positive correlation between MLR and MRS scores (p < 0.001). Univariate regression analysis was applied to study whether high MLR was associated with poor prognosis, and we found that MLR increased with the 1-year outcome, with the OR value of 5.919 (95% CI, 1.279–27.396; p = 0.023). Age (p = 0.031), history of hyperlipidemia (p = 0.014), and albumin level (p = 0.012) also had a meaningful relationship with poor prognosis (Table 4). Based on the above-mentioned, we established two models: model 1 contained these three parameters, and model 2 included MLR in addition to the above-mentioned parameters (Table 5). We used the area under the ROC curve (AUC) to analyze the predicted values of the two models and found that the AUC value of model 1 was 0.685, and that of model 2 was 0.726. The AUC of the BI score as the second diagnostic prognostic criterion was 0.847 (Figure 4). In DeLong analysis, there were also differences in ROC curves between the two models (p = 0.0367). Therefore, in the development group, MLR had predictive value for the prognosis of LAA cerebral infarction. In the validation cohort, we also made a model that included age, history of hyperlipidemia, albumin level, and MLR. Then, we found that the AUC of this prediction model was 0.857 when the AUC of the BI was 0.889, which also predicted the prognosis well (Figure 4).

**Table 4:** Univariate logistic regression analyses for prognosis in the development cohort.

	1-year outcome	
	Hazard ratio (95% CI)	p-value∗
NIHSS	2.006 (1.615–2.493)	<0.001∗
ASPECTS	0.493 (0.0378–0.643)	<0.001∗
Age	1.031 (1.003–1.063)	0.031∗
Hyperlipidemia	0.424 (0.214–0.842)	0.014∗
HP	3.435 (0.996–11.842)	0.051
Neutrophil	1.319 (1.107–1.573)	0.002∗
Lymphocyte	0.405 (0.224–0.730)	0.003∗
Monocyte	14.179 (2.629–76.460)	0.002∗
Albumin	0.880 (0.796–0.973)	0.012∗
TC	0.752 (0.551–1.027)	0.073
MLR	5.919 (1.279–27.396)	0.023∗

Note: Use the univariate logistic regression analyses. Abbreviation: NIHSS: the National Institutes of Health Stroke Scale; HP: hypertension; TC: total cholesterol; MLR: monocyte-to-lymphocytes ratio. (∗p < 0.05).

**Table 5:** Univariate analyses for the potential factors associated with Prognosis of 1-year LAA cerebral infarction by Logistic regression in development cohort.

Model 1				Model 2			
	P	OR	95%CI		P	OR	95%CI
Age	0.192	1.020	0.990–1.051	Age	0.286	1.017	0.986–1.048
HL	0.030∗	2.174	1.080–4.375	HL	0.028∗	2.222	1.088–4.539
ALB	0.061	0.904	0.813–1.005	ALB	0.075	0.908	0.816–1.010
				MLR	0.048∗	4.037	1.013–16.088

Note: Logistic regression analysis found that patients' Age, Hyperlipidemia, and albumin are independent risk factors for LAA cerebral infarction. On this basis, we have established two models, and Model 2 add MLR.

Abbreviation: HL: Hyperlipidemia; ALB: Albumin; MLR: monocyte-to-lymphocytes ratio. (∗p < 0.05).

### 3.4 Diagnostic efficacy of MLR in LAA in the development cohort

**Figure 5:**
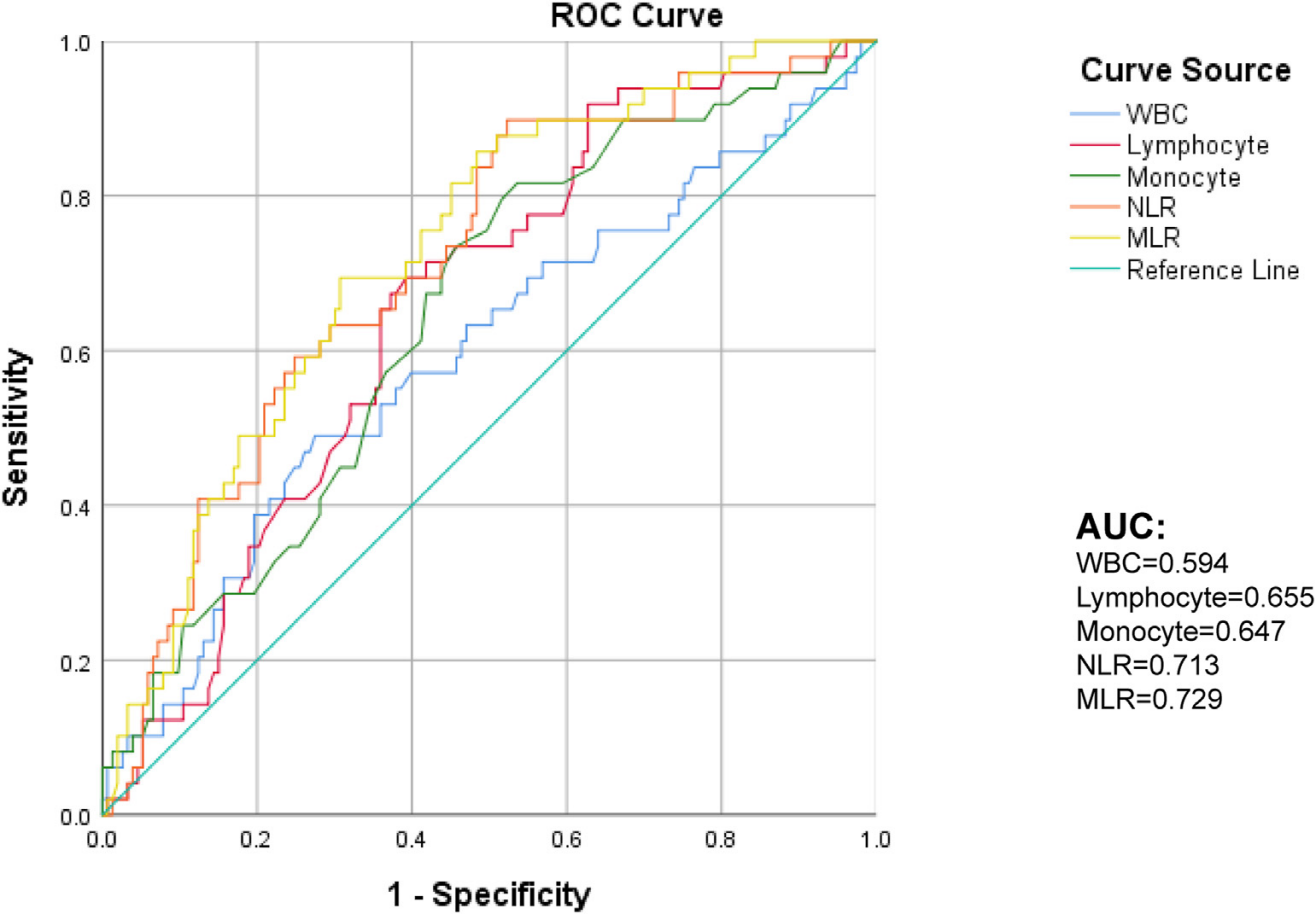
The receiver operating characteristic curve of neutrophils, lymphocytes, and white blood cell counts, MLR, and NLR predicting values of the 1-year outcome in LAA cerebral infarction patients. MLR, monocyte to lymphocyte ratio; NLR, neutrophil to lymphocyte ratio; ROC, receiver operating characteristics.

According to ROC curve analysis, the optimal critical value of MLR for the prediction of LAA-type stroke was 0.34. The AUC of MLR was 0.729 by computing (95% CI, 0.653–0.86, p < 0.001), as it had a sensitivity of 69.3% and a specificity of 69.4% (Figure 5). In the binary logistics regression analysis, WBC (p = 0.024), lymphocyte (P = 0.003), and monocyte (p = 0.002) counts were indicators with significance for one-year prognosis (Table 4). The AUC of MLR against WBC count, as shown in Figure 5, was 0.594 (95% CI, 0.499–0.690, p = 0.047), which had a sensitivity of 49.0% and a specificity of 72.5%. The AUC of neutrophil to lymphocyte ratio (NLR) was 0.713 by calculating (95% CI, 0.633–0.793, P < 0.001). Therefore, MLR showed better performance in predicting the prognosis of LAA stroke (AUC = 0.729, P < 0.001) (Figure 5). We also used the DeLong test to analyze the pairwise comparison of ROC curves and found that the predicted value of the ROC curve of MLR was different from other single indicators (p < 0.05). The ROC curve prediction value of NLR was only different from WBC (p = 0.016). Hence, MLR had better prognostic performance than individual WBC, lymphocyte, and monocyte counts and NLR markers (Supplementary materials).

### 3.5 Investigating factors associated with MLR in the development cohort

**Figure 6:**
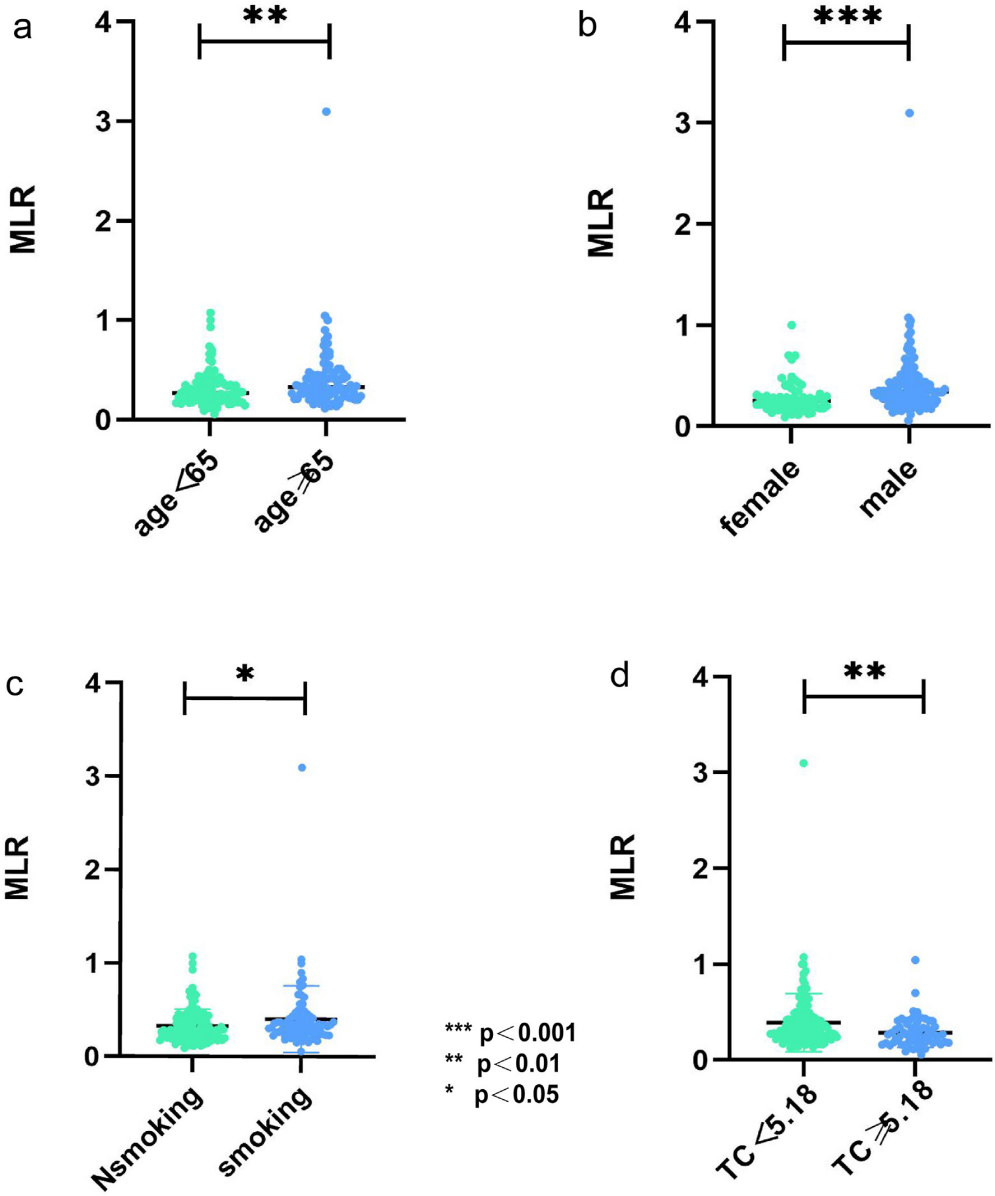
The relationship between MLR and age, gender, smoking, and TC. (∗∗∗p < 0.001; ∗∗p < 0.01; ∗p < 0.05) TC, total cholesterol.

As shown in Figure 6, patients older than 65 years had higher MLR than those younger than 65 years (p = 0.003) (Figure 6a). We found that MLR in women was lower than that in men (P < 0.001) (Figure 6b). MLR was higher in smoking patients than in non-smoking patients (p = 0.03) (Figure 6c). MLR with TC of <5.18 was higher than that in hypercholesterolemia patients (p = 0.003) (Figure 6d). Meanwhile, further study showed that there was a significant correlation between MLR and age (r = 0.269, P < 0.001), TC (r = −0.199, p = 0.005); smoking (r = 0.153, p = 0.030), and gender (r = 0.313, P < 0.001).

## 4 Discussion

MLR, as a simple ratio between monocytes and lymphocytes, is a recent novel biomarker of the inflammatory response, and the relationship between MLR and the prognosis of LAA-type stroke has not been reported yet. To the best of our knowledge, our research is the first one to investigate whether there is a connection between MLR and the prognosis of LAA-type cerebral infarction.

With the advancement of imaging, molecular biology, and laboratory science, the diagnosis of LAA-type cerebral infarction is currently not difficult [27, 28]. However, the prognosis of cerebral infarction is still challenging because of the limited predictive properties of existing models, and our study provided a new idea for prognosis estimation. We hoped to find a direct and effective index to combine clinical scores to increase the evaluation power of the prognosis of LAA-type cerebral infarction patients. The inflammatory response after stroke has been widely considered to be related to the evolution of infarction [29]. At the same time, atherosclerosis is characterized by chronic continuous inflammation of arteries, which is, in turn, characterized by immune cell infiltration [30]. As the two main participants of the inflammatory response, different subtypes of WBCs are considered to exacerbate the complex situation of secondary brain injury after the onset of LAA cerebral infarction [31]. NLR and MLR are the most commonly studied inflammatory composite indicators. In previous studies, more attention has been paid to NLR. Both increased neutrophils and decreased lymphocytes exacerbate oxidative damage to the vessel wall. In a study involving 1233 patients with AIS, NLR has been associated with the hemorrhagic transformation of AIS [32]. In one study, MLR has also been associated with the progression of carotid atherosclerosis [33]. Thus, NLR and MLR are considered to be of research value. However, in our study, we found that MLR had a more significant effect on the prognosis of patients with LAA-type cerebral infarction than NLR.

Therefore, the integration of monocytes and lymphocytes into a single index by MLR might be a better predictor of the prognosis of LAA-type stroke patients. In our study, higher MLR at admission had a close association with severe stroke in LAA-type AIS patients. Concurrently, in the establishment of both development and validation regression models, we found that MLR was significantly associated with poor prognosis in LAA infarction patients, which was consistent with the results of stroke severity, and poor outcome was closely associated with lower LMR found by Hao Ren et al. [34]. We also analyzed the predictive values of WBC, lymphocyte, and monocyte counts and NLR as inflammatory indicators for the prognosis of LAA through the Delong test to better compare the prediction of MLR. We found that compared with the inflammatory indicators mentioned above, the predictive value of MLR was higher. Hence, in our study, MLR was a better predictor. Finally, we analyzed the relationship between basic information and MLR. We found that gender and age had significant effects on MLR. With the increase in age, MLR gradually increased. Men had higher MLR than women, and this has also been found in the study by Meng X et al. [35]. This is consistent with a finding of a positive correlation between MLR and age and gender in the study by Gao et al. [36]. It has also been confirmed by Ekicy et al. that MLR was lower in the non-smoking population than in the smoking population [37]. This is mainly due to systemic oxidant-antioxidant imbalance caused by long-term smoking and the related obvious low-grade inflammatory response [38].

Inflammation plays an important role in cerebral infarction. Stroke mobilizes immature pro-inflammatory Ly6C^hi^CD43^lo^ monocytes, which rapidly infiltrate into ischemic tissue and reach the core of the lesion [39]. The generation of tumor necrosis factor (TNF)-α in monocytes has been independently associated with stroke [40]. Lymphocyte count is considered to have a neuroprotective effect, improving cognitive function [41]. After a stroke, MLR might increase due to the increase in monocytes and the decrease in lymphocytes caused by the activation of the sympathetic nervous system and hypothalamic-pituitary-adrenal system [42]. As an important inflammatory marker, MLR is associated with the occurrence and prognosis of many diseases, such as vitiligo [43], COVID-19 [15], cancer [14], etc. Similar to ischemic stroke, inflammatory mechanisms also play an important role in the occurrence and prognosis of these diseases. Therefore, we reasoned that MLR might also be related to the prognosis of AIS. Previous studies have also shown that increased MLR was considered an independent risk factor and represented a peripheral marker in patients with ischemic stroke [44, 45]. At the same time, MLR has been associated with cardioembolic stroke [46], post-stroke depression [47], and carotid stenosis in ischemic stroke [33]. Therefore, the MLR index has great clinical application value. In our study, high MLR was associated with poor prognosis in LAA stroke. Additionally, the area under the ROC curve of the predictive value of BI and MLR for the prognosis of LAA cerebral infarction only slightly differed. This suggests that we should pay attention to MLR in the early stage of LAA stroke disease to improve the prognosis of patients.

Our study had some limitations. First, the study population and sample size were limited, and this study had internal validation but lacked external validation. In the following research, we will verify the model accuracy by using the method of multicenter cooperation for external verification. Second, the relationship between MLR and prognosis in AIS patients might have been affected because MLR values were only collected at admission and had no dynamic detection. We could not clearly explain the specific mechanism of the prognostic effect of MLR in patients with LAA. Thus, it needs more experimental research. However, our preliminary research can also provide meaningful direction in clinical practice.

## 5 Conclusions

Our study suggests that high MLR levels in LAA-type AIS patients are associated with poor prognosis. Meanwhile, age, gender, and smoking are also related to MLR. The high level of early MLR suggests that clinicians should pay more attention to LAA-type AIS patients’ condition to improve their prognosis.

## Declarations

### Author contribution statement

Cheng-ju Wang, Chun-yang Pang, Huan-jie Huang: Conceived and designed the experiments; Performed the experiments; Analyzed and interpreted the data; Contributed reagents, materials, analysis tools or data; Wrote the paper.

Huan-Yu, Yi-fan Cheng, Hong Wang, Bin-bin Deng: Analyzed and interpreted the data; Contributed reagents, materials, analysis tools or data; Wrote the paper.

### Funding statement

This research did not receive any specific grant from funding agencies in the public, commercial, or not-for-profit sectors.

### Data availability statement

Data will be made available on request.

### Declaration of interest's statement

The authors declare no conflict of interest.

### Additional information

No additional information is available for this paper.

## Appendix A Supplementary data

Supplementary materials.docx Supplementary materials.docx

CWS_Editorial_Certificate.pdf CWS_Editorial_Certificate.pdf
