# Corrigendum to “Monocyte-to-lymphocyte ratio affects prognosis in LAA-type stroke patients” [Heliyon Volume 8, Issue 10, October 2022, Article e10948]

**DOI:** 10.1016/j.heliyon.2025.e43340

**Published:** 2025-04-22

**Authors:** Cheng-ju Wang, Chun-yang Pang, Huan-Yu, Yi-fan Cheng, Hong Wang, Bin-bin Deng, Huan-jie Huang

**Affiliations:** The First Affiliated Hospital of Wenzhou Medical University, Wenzhou, China

In this article, the authors omitted a complete statement in their article regarding ethical approval for the research presented in this publication, which is required under the policies of the journal. The authors confirm that ethical approval was obtained from Ethics Committee in Clinical Research (ECCR) of the First Affiliated Hospital of Wenzhou Medical University for the research under the following reference number 2017-R008.

*The original Ethical approval statement in section 2.1*. Patient selection is as below.

The Medical Ethics Committee of the First Affiliated Hospital of Wenzhou Medical University approved this research.

To fix this issue, the sentence should be updated with the complete ethical committee and approval number.

The correct version of *Ethical approval statement* should be as below.

*This study was reviewed and approved by the Ethics Committee in Clinical Research (ECCR) of the First Affiliated Hospital of Wenzhou Medical University (Approval No. 2017-R008)*.

The authors apologize for the errors.

## Declaration of competing interest

The authors declare no conflict of interest.

