# Supplementary material for: Monocyte-to-lymphocyte ratio affects prognosis in LAA-type stroke patients
Source: Heliyon. 2022 Oct 4;8(10):e10948. doi: 10.1016/j.heliyon.2022.e10948 (PMC9561738; doi:10.1016/j.heliyon.2022.e10948)
Supplement: Supplementary materials.docx [file mmc1.docx]

Supplementary materials: Pairwise comparison of ROC curves

|  | Difference between areas | 95% Confidence Interval | p |
| --- | --- | --- | --- |
| Monocyte-NLR | 0.066 | 0.052(-0.036-0.168) | 0.202 |
| WBC-NLR | 0.119 | 0.049(0.022-0.215) | 0.016* |
| Lymphocyte-NLR | 0.059 | 0.034(-0.0075-0.125) | 0.083 |
| Monocyte-MLR | 0.083 | 0.038(0.0076-0.158) | 0.031* |
| WBC-MLR | 0.135 | 0.053(0.0304-0.240) | 0.011* |
| Lymphocyte-MLR | 0.075 | 0.036(0.0048-0.145) | 0.036* |
| WBC-Lymphocyte | 0.060 | 0.073(-0.083-0.203) | 0.408 |
| WBC-Monocyte | 0.052 | 0.036(-0.017-0.122) | 0.141 |
| Monocyte-Lymphocyte | 0.0078 | 0.067(-0.122-0.138) | 0.907 |
| NLR-MLR | 0.016 | 0.029(-0.041-0.074) | 0.575 |

Use Delong test statistical method. NLR: neutrophil-to-lymphocyte ratio, MLR: monocyte-to-lymphocytes ratio. (*p < 0.05)
