# Supplementary material for: Monocyte-to-lymphocyte ratio affects prognosis in LAA-type stroke patients
Source: Heliyon. 2022 Oct 4;8(10):e10948. doi: 10.1016/j.heliyon.2022.e10948 (PMC9561738; doi:10.1016/j.heliyon.2022.e10948)
Supplement: CWS_Editorial_Certificate.pdf [file mmc2.pdf]

Charlesworth Author Services

EDITORIAL CERTIFICATE

This document certifies that the manuscript below was edited for correct English language usage, grammar, punctuation and spelling by qualified native English speaking editors at Charlesworth Author Services.

Paper Title:

Monocyte-to-lymphocyte Ratio affects prognosis in LAA-Type Stroke Patients

Author:

王 程菊

Date certificate issued:

August 18, 2022

cwauthors.com
